# Tuberculosis in Kindergarten and Primary School, Italy, 2008–2009

**DOI:** 10.3201/eid1703.101440

**Published:** 2011-03

**Authors:** Antonietta Filia, Giuseppe Ciarrocchi, Rossana Belfiglio, Monaldo Caferri, Antonino Bella, Claudio Piersimoni, Daniela Cirillo, Gualtiero Grilli, Cristina Mancini, Donato Greco

**Affiliations:** Author affiliations: National Health Institute, Rome, Italy (A. Filia, A. Bella, D. Greco);; Local Health Authority Marche Region, Ancona, Italy (G. Ciarrocchi, R. Belfiglio, M. Caferri);; United Hospitals, Ancona (C. Piersimoni);; Regional Reference Mycobacterial Laboratory, Ancona (C. Piersimoni);; San Raffaele Scientific Institute, Milan, Italy (D. Cirillo);; Regional Healthcare Agency Marche Region, Ancona (G. Grilli, C. Mancini)

**Keywords:** Tuberculosis, tuberculosis and other mycobacteria, bacteria, outbreak, kindergarten, primary school, children, Italy, dispatch

## Abstract

An outbreak of tuberculosis (TB) in Italy involved 19 schoolchildren with active TB and 43 with latent infection. The source of the outbreak was a school assistant born in Italy who had a family history of TB. This outbreak highlights the need for maintaining clinical and public health expertise in countries with low TB incidence.

The decrease in incidence of tuberculosis (TB) since the mid-1900s in Italy has resulted in loss of expertise in TB diagnosis, management, and control (*1*). However, TB is still present in Italy; each year >4,000 new cases are reported to the statutory reporting system (7.4 cases/100,000 population in 2008), and 46% of cases occur in foreign-born persons (*2*). As in other industrialized countries, cases in children are uncommon. Occurrence of TB in children indicates ongoing transmission of the disease and reflects effectiveness of a TB control program. Several TB outbreaks in schools have been reported in low-incidence countries in the past decade (*3**–**6*). We report an outbreak that occurred in a kindergarten/primary school in Italy.

## The Study

A contact investigation was performed by local health authorities in central Italy after a report in November 2008 of culture-positive pulmonary TB in a 3-year-old girl. She attended a kindergarten in a building that housed 3 schools (kindergarten 1, kindergarten 2, and a primary school). Initially, children in the girl’s class (kindergarten 1, class C), her family contacts, and some staff were tested. A high percentage (34.6%, 9/26) of classmates were positive for TB by Mantoux tuberculin skin test (TST), and health authorities decided to test all school children and staff. Contacts of persons with new cases identified were also tested. This contact investigation was part of a routine public health response to a school outbreak and did not require ethics committee approval.

Persons were screened by clinical evaluation, TST, and/or chest radiography. A positive TST result was defined as induration >5 mm (*7*). Persons with negative TST results were retested 10 weeks later. Children with positive TST results for whom a diagnosis of active TB had been ruled out were prescribed isoniazid for 6 months. Children <5 years of age were prescribed isoniazid regardless of TST results if active TB was ruled out. Those children with negative results at initial screening stopped treatment after 10 weeks if repeated testing showed negative results. Persons with chest radiograph abnormalities compatible with TB underwent further diagnostic evaluation.

A total of 851 persons, of whom 817 (96%) were born in Italy, were screened (Table 1). At initial testing, 53 (13.8%) of 383 children had positive TST results. Among 9 children with a positive result at follow-up, 1 had not undergone initial testing; 8 children showed skin test result conversion. Nineteen children had active TB (18 with pulmonary TB and 1 with extrapulmonary TB [Pott disease]). Forty-three children had latent TB infection (Table 2). No cases of active TB were identified among school staff and contacts of children with positive TST results.

**Table 1:** Results of initial and follow-up screening for tuberculosis, Italy, 2008–2009*

Persons screened by TST or chest radiograph	No. tested	Median age, y (range)	TST induration >5 mm, no. positive/no. tested (%)		No. positive/no. with negative TST result at initial screening (%)
			Initial screening	Follow-up testing	
Schoolchildren	388	6 (2–11)	53/383 (13.8)	9/320 (2.8)	320/330 (97.0)
School staff	77	45 (24–60)	15/71 (21.1)	7/53 (13.2)	53/56 (94.6)
Family contacts†	207	37 (1–89)	35/203 (17.2)	10/90 (11.1)	90/168 (53.6)
Casual contacts	179	29 (2–66)	18/173 (10.4)	4/106 (3.8)	106/155 (68.4)
Total	851	8 (1–89)	121/830 (14.6)	30/569 (5.3)	569/709 (80.2)

*All categories are mutually exclusive. TST, tuberculin skin test. †Household and immediate family members of children with positive skin test results. Does not include family contacts of the source case-patient.

**Table 2:** Attack rates for of children with latent and active tuberculosis in 2 kindergartens and primary school, Italy, 2008–2009*

School class and grade	No. students	Median age, y (range)	No. latent TB/no. tested (%)	No. active TB/no. tested (%)	No. infected/no. tested (%)
Kindergarten 1	107	4 (3–6)	16/103 (15.5)	13/103 (12.6)	29/103 (28.2)†
Class A	23	4 (4–6)	2/22 (9.1)	4/22 (18.2)	6/22 (27.3)
Class B	28	5 (4–6)	7/27 (25.9)	4/27 (14.8)	11/27 (40.7)
Class C	28	4 (3–4)	5/26 (19.2)	4/26 (15.4)	9/26 (34.6)
Class D	28	3 (3–3)	2/28 (7.1)	1/28 (3.6)	3/28 (10.7)
Kindergarten 2	82	4 (2–6)	3/81 (3.7)	3/76 (3.9)	6/81 (7.4)
Primary school	199	8 (5–11)	24/199 (12.1)	3/192 (1.6)	27/199 (13.6)
Grade 1	45	6 (5–8)	16/45 (35.6)	3/45 (6.7)	19/45 (42.2)†
Grade 2	29	7 (6–7)	3/29 (10.3)	0/26 (0.0)	3/29 (10.3)
Grade 3	40	8 (7–9)	1/40 (2.5)	0/38 (0.0)	1/40 (2.5)
Grade 4	47	9 (9–11)	2/47 (4.3)	0/47 (0.0)	2/47 (4.3)
Grade 5	38	10 (9–10)	2/38 (5.3)	0/36 (0.0)	2/38 (5.3)
Total	388	6 (2–11)	43/383 (11.2)	19/371 (5.1)	62/383 (16.2)

*TB, tuberculosis. †p<0.001 by χ^2^ test or, when appropriate, Fisher exact test or χ^2^ test for trend.

In January 2009, a case of smear-negative, culture-positive pulmonary TB with cavitary involvement of both upper lung lobes was reported to the same local health authorities by a hospital in southern Italy. The case-patient was a 42-year-old woman born in Italy who worked as an assistant in kindergarten 1 and was considered the presumed source of the outbreak. She reported a family history of TB (son and father treated for pulmonary TB in 1996 and 1999, respectively) and positive TST results, but did not recall completing a chemoprophylaxis regimen with isoniazid. She also reported persistent cough for 1 year, which was treated with mucolytic and antimicrobial drugs by her family physician. During this time, she had frequent contact with many children, especially in kindergarten 1, and occasionally worked as an assistant in kindergarten 2. She had a negative TST result during the school contact investigation; no chest radiograph was prescribed. She subsequently traveled to southern Italy, where she consulted a respiratory physician who requested immediate hospitalization.

Gastric aspirate specimens were obtained from 18 children, and a vertebral abscess pus specimen was obtained from 1 child. Sputum specimens were obtained from the source case-patient. Ziehl-Neelsen microscopy, direct amplification tests, cultures, and drug-susceptibility testing were performed according to standard procedures. Specimens from 3 children were positive for acid-fast bacilli, and specimens from 9 children were culture positive for *Mycobacterium tuberculosis*. All isolates were susceptible to first-line drugs against TB except 1 that was moderately resistant to isoniazid. DNA fingerprinting showed that the strain isolated from the source case-patient belonged to the S family lineage and was identical to all strains isolated from children in this study.

Median age of children with active TB was 5 years (9 were girls and 15 had signs or symptoms). Attack rates were significantly higher for children in kindergarten 1 than for those in kindergarten 2 and the primary school (p<0.001) (Table 2). Differences in attack rates in 4 classes of kindergarten 1 were not significant. In the primary school, high attack rates were found among children in grade 1; no active TB cases and few children with positive TSTs results were found in higher grades (p<0.001). Most children in grade 1, including the 3 with active TB, were in kindergarten 1 the preceding school year. All children except 1 (lost to follow-up) have completed treatment and are healthy.

## Conclusions

This outbreak indicates TB transmission in a low-incidence country. Genotyping results confirmed that all cases were linked to the school assistant, and clinical and epidemiologic data suggest that she was the only source of infection. Infected children in grade 1 were most likely infected the preceding school year. Young children with TB are not usually infectious. During this outbreak, it is unlikely that they transmitted TB to others. The major factor that contributed to the outbreak was the delay in diagnosis of the source case, as in other reported outbreaks (*3**–**6*). This finding indicates the need for increasing awareness of TB among primary care physicians and for improving their ability to recognize risk factors for infection and progression to active disease. Also, if the school assistant had received appropriate treatment and follow-up at the times her son and father received diagnoses of TB, active disease might never have developed. This finding highlights the importance of appropriate follow-up of contacts in response to a case of TB.

Our study has several limitations. Urgency was required because of the school setting, pressure by parents, and media coverage. As in other school-contact investigations, this urgency may have led to excessive screening of low-risk persons and difficulties in categorizing contacts by close and casual status (*8*). Also, information regarding risk factors for TB and previous TST results was not consistently recorded. Nevertheless, the investigation had a high rate of test completion among children and staff.

Although TB among foreign-born persons is of increasing importance in many industrialized countries because of immigration from high-incidence areas, this outbreak highlights that TB is of concern not only for those of foreign origin (*2*). TB was once a common disease in Italy, and a pool of improperly treated persons may be at risk for reactivation of TB. A high proportion of school staff had positive TST results, probably because many were born at a time when the risk for TB was high. In Italy, there are no specific requirements regarding TB screening of school employees. When screening is initiated in a low-prevalence population, it should focus on high-risk persons, and priority should be given to quality surveillance, follow-up, and containment activities. However, consideration should be given to administering a health questionnaire to school staff (foreign and native born) who have sustained contact with small children to identify high-risk persons, as is being conducted elsewhere in Europe (*3**,**9*).
